# The Viral Hypothesis in Alzheimer’s Disease: Novel Insights and Pathogen-Based Biomarkers

**DOI:** 10.3390/jpm10030074

**Published:** 2020-07-29

**Authors:** Sean X Naughton, Urdhva Raval, Giulio M. Pasinetti

**Affiliations:** 1 Department of Neurology, Icahn School of Medicine at Mount Sinai, New York, NY 10029, USA; sean.naughton@mssm.edu (S.X.N.); Urdhva.Raval@mssm.edu (U.R.); 2 James J. Peters Veterans Affairs Medical Center, Bronx, NY 10468, USA

**Keywords:** Alzheimer’s disease, virus, bacteria, dementia

## Abstract

Early diagnosis of Alzheimer’s disease (AD) and the identification of significant risk factors are necessary to better understand disease progression, and to develop intervention-based therapies prior to significant neurodegeneration. There is thus a critical need to establish biomarkers which can predict the risk of developing AD before the onset of cognitive decline. A number of studies have indicated that exposure to various microbial pathogens can accelerate AD pathology. Additionally, several studies have indicated that amyloid-β possess antimicrobial properties and may act in response to infection as a part of the innate immune system. These findings have led some to speculate that certain types of infections may play a significant role in AD pathogenesis. In this review, we will provide an overview of studies which suggest pathogen involvement in AD. Additionally, we will discuss a number of pathogen-associated biomarkers which may be effective in establishing AD risk. Infections that increase the risk of AD represent a modifiable risk factor which can be treated with therapeutic intervention. Pathogen-based biomarkers may thus be a valuable tool for evaluating and decreasing AD risk across the population.

## 1. Introduction

Alzheimer’s disease (AD) is an age-related neurodegenerative disorder. AD results in progressive cognitive decline, and is the most common form of dementia in older adults. This incurable disorder is predicted to affect approximately 100 million people globally by 2050 [1,2]. The characteristic hallmarks of AD pathology are amyloid-beta (Aβ) peptide plaques, tau hyperphosphorylation, and neuroinflammation. Currently, a clinical diagnosis of AD is only possible after disease onset through post-mortem detection of amyloid plaques and neurofibrillary tangles [3]. Cognitive testing can aid in the diagnosis of dementia, but strong cognitive impairments are usually only present at a time point when successful therapeutic intervention is unlikely. Early diagnosis of AD is only possible in rare cases in which the autosomal dominant early onset form of the disease is genetically inherited [4]. Considering the impact and prevalence of AD globally, there is an increasing need to understand and identify biomarkers, in order to detect AD in individuals before the onset of disease and provide mitigating therapeutics. Preclinical detection of biomarkers of Aβ, tau, and other neurodegenerative effects have been extensively studied. Aβ and tau have been detected in cerebrospinal fluid and blood plasma. Neuroimaging via magnetic resonance imaging (MRI) and positron emission tomography (PET), specifically, FDG-PET, amyloid PET and structural MRI can also serve diagnostic roles in AD [1,5]. Interestingly, there is some indication of microbial and viral involvement in AD pathology. Given the relatively high prevalence of certain pathogens, they can serve as biomarkers for preclinical AD. In this review, we explore the role of microbes in AD pathology and the potential of various pathogens as novel biomarkers for AD. This review is based on literature generated from searches conducted between 1 April and 25 July 2020, using standard databases and search engines for scientific literature (PubMed and Google Scholar), using the following keywords: “Alzheimer’s Disease”, “Pathogen Hypothesis” “Viruses”, “Bacteria”. Additional references were collected from those discussed in the literature generated through the search.

### Amyloid-Beta

The Aβ peptide is integral to AD pathology. Aβ misfolding and the resultant Aβ plaques are thought to be the root cause of cognitive decline in AD. Aβ aggregates are formed from the proteolytic cleavage of a larger type 1 membrane glycoprotein, named amyloid precursor protein (APP). APP is involved in maintaining neuronal homeostasis, neuronal development, signaling, and intracellular transport [6]. APP is cleaved by β-secretases and γ-secretases, to produce an Aβ peptide ranging from 37 to 49 amino acid residues [7]. Aβ aggregates are found in the hippocampus, neocortex, and cerebrovasculature [8]. Aβ exists in different forms, including soluble Aβ, Aβ oligomers, and Aβ plaque forms. These different forms are involved in neurodegeneration at different stages of AD [6]. Aβ plaques induce tau protein hyperphosphorylation and formation neurofibrillary tangles and synaptic dysfunction. These plaques also generate the production of 4-hydroxynonenal, a toxic aldehyde involved in lipid peroxidation, and disruption of cellular homeostasis [9]. Aβ aggregation also leads to DNA damage and the release of inflammatory responses which result in the loss of neuronal synapses and ultimately neuronal death [10]. Intriguingly, Aβ acts as an antimicrobial peptide (AMP) and has been demonstrated to be effective against viruses, bacteria, and fungi. AMPs are a group of defensins, histatins, and cathelicidins that primarily serve to defend the host against a wide variety of pathogens. AMPs can also modulate cytokine release and adaptive immune responses. Aβ has been demonstrated to function like the cathelicidin AMP LL-37. Aβ was shown to be effective against the bacteria Streptococcus pneumoniae and fungus Candida albicans, which are the causative agents of bacterial meningitis and neurocandidiasis, respectively. Aβ also inhibits certain other bacterial species of the genera Pseudomonas, Escherichia, Streptococcus, Staphylococcus, Salmonella, and Enterococcus [11,12]. There is also evidence indicating that Aβ can also inhibit replication of seasonal and pandemic strains of the influenza A and herpes simplex virus 1 (HSV-1) viruses [13]. In fact, Aβ has been shown to be as effective as the antiviral drug Acyclovir at inhibiting HSV-1 neuropathology [14].

## 2. Pathogens and AD

### 2.1. Viral Pathogens in Neurodegeneration and AD

**Figure 1:**
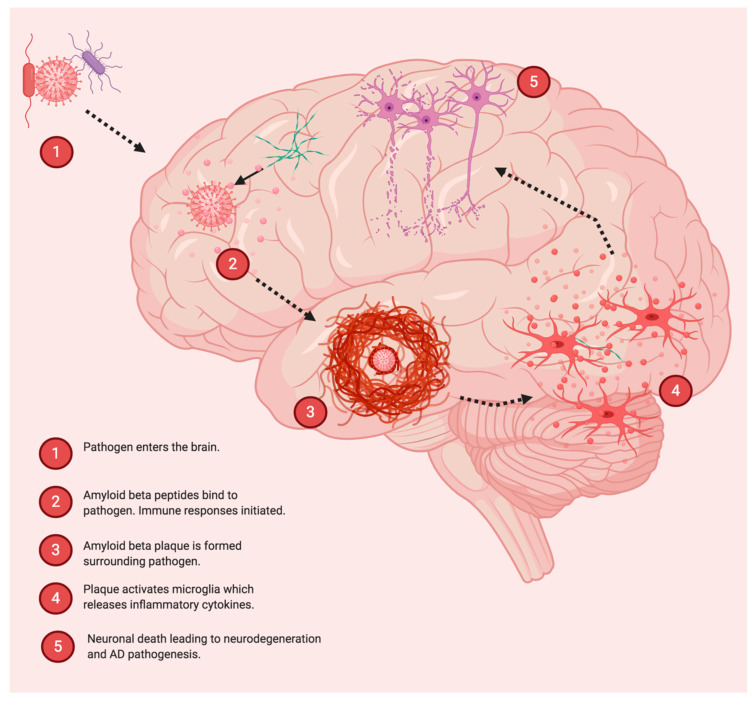
Involvement of Pathogens in Alzheimer’s disease (AD). Pathogens such as viruses and bacteria can become entrapped by amyloid-β after entering the brain. Amyloid fibrils form in response to certain pathogens, and infection may play a role in accelerating AD pathology by stimulating inflammation and neurodegeneration.

The idea that infections may play a role in Alzheimer’s disease (AD) pathogenesis dates back nearly 30 years and has been a subject of debate in the field of AD (Figure 1) [15]. Previous studies have suggested that amyloid-β (Aβ) may act as a part of the innate immune system to aggregate around infectious particles. Eimer and colleagues showed that 5XFAD mice infected with herpes simplex virus 1 (HSV-1) showed increased survival rates compared to infected non-transgenic littermates. Moreover, Aβ was found to bind to and entrap HSV1, in a process mediated by fibrillization. Aβ deposition could be triggered by HSV1 infection in young 5XFAD mice, prior to the ordinary development of Aβ deposits [16]. Additionally, brains from Alzheimer’s disease patients have been shown to have increased levels of human herpesvirus 6 and human herpesvirus 7 in several key areas [17]. HSV-1 infection has also been shown to drive the development of amyloid fibrillar plaque-like formations in human-induced neural stem cells and 3D human brain-like tissue cultures [18]. While different types of herpesvirus have been associated with AD pathology and detected in the brains of AD patients, there is also evidence to suggest that other viruses (and other types of pathogens) may also play a role in AD. For example, Nimgaonkar and colleagues found that exposure to HSV-2, cytomegalovirus (CMV), or the parasite Toxoplasma gondii (TOX) was associated with cognitive decline in individuals aged 65 and older [19]. Additionally, Ljungan virus (LV) has been detected in the hippocampus of AD brains, but not in age-matched controls [20]. The detection of different viral strains in the brains of different cohorts of patients hints at the idea that viral infection may play a role in AD or that AD may increase susceptibility to neuroinvasion by viruses. It is currently unclear if one or more pathogenic infections might directly stimulate (or accelerate) AD, or if AD creates an environment which facilitates the accumulation of infections in the brain through altered immune function. In this regard, it is interesting to consider the case of HIV, in which immune function is compromised in the presence of a persistent viral infection. In particular, HIV-associated neurocognitive disorders (HAND) have been associated with the presence of β-amyloid, and HAND patients have been shown to have similar cerebrospinal fluid levels of β-amyloid 1-42 when compared to patients with Alzheimer’s associated dementia [21,22] Compromised immune function may thus be a critical driver of neurodegeneration by allowing infectious pathogens such as viruses to enter the brain at levels which exceed the capacity of the innate immune system within the brain. A number of different viruses have been shown to enter the brain and cause neurodegeneration, often with accompanying protienopathy. For example, the family of H5N1 avian influenza A viruses responsible for a previous epidemic in Asia have been shown to produce Parkinsonian-like neurodegeneration in mice. H5N1 induced neurodegeneration was accompanied by ⍺-synuclein phosphorylation and neuroinflammation. Interestingly, microgliosis persisted long after the infection resolved and was observable at a time point 90 days from the initial infection [23].

An intriguing idea which has emerged regarding viral infection and neurodegeneration is the “multi-hit” or “hit and run” hypothesis. This notion is supported by a study from Sadasivan and colleagues, who showed that infection with influenza H1N1 virus 30 days prior to MPTP administration markedly enhanced neurodegeneration in the substantia nigra pars compacta (SNpc) of mice. Furthermore, this enhancement of MPTP induced neurodegeneration could be alleviated by prior vaccination [24]. Thus, it is possible that a viral infection at one time point may later synergize with other factors (i.e., environmental toxins, lifestyle choices, genetic background), to cause or accelerate neurodegeneration. The above-mentioned studies regarding influenza viruses and Parkinsonian like neurodegeneration provide additional insight as to the potential role of viral pathogens in stimulating neurodegeneration independent of β-amyloid. Persistent inflammation stimulated by viral infection may be a critical component in predisposing individuals to AD. Neuroinflammation and neuro-immune interactions have gained attention in recent years as potential driving factors of neurodegeneration [25]. Thus, the pathological effects of increased neuroinflammation paired with its ability to inhibit amyloid clearance may create a bi-directional relationship, whereby viruses (or other pathogens) are able to both increase amyloid activity through direct interactions, while preventing amyloid clearance through stimulation of inflammation.

### 2.2. Bacterial Pathogens and AD

**Figure 2:**
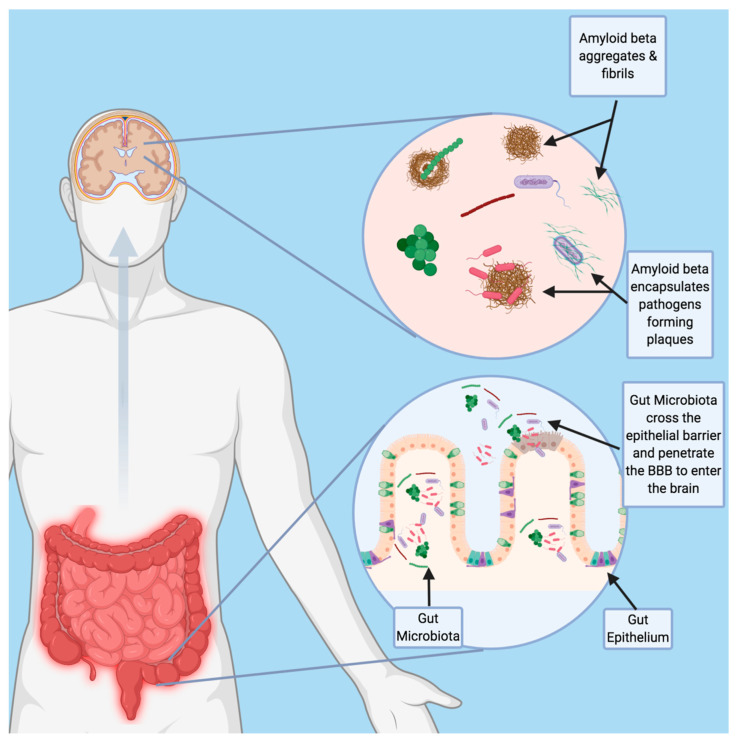
Potential Role of Gut Bacteria in Neurodegeneration. Age-related changes in intestinal permeability and blood brain barrier integrity may allow for penetrance of gut bacteria into the brain and promote the formation of amyloid fibrils. Future studies should focus on the role of gut bacteria as a potential trigger to the amyloid cascade.

In addition to the above-mentioned viruses, bacterial pathogens have also been associated with AD. In particular, there is mounting evidence linking periodontal disease and AD. Periodontitis, commonly known as gum disease, is an oral infection resulting in the release of proinflammatory cytokines into the bloodstream and the increase of C-reactive protein. It is caused by the gram-negative anaerobic bacterium Porphyromonas gingivalis [26]. P. gingivalis and its associated toxins, referred to collectively as gingipains, have been identified in 96% of postmortem brain tissue samples of AD patients and are thought to exacerbate AD pathology [27]. Gingipains play a key role in P. gingivalis mediated aggravation of AD. Gingipains are a group of cysteine proteases secreted by P. gingivalis that cause neuronal damage, increased tau production, and increased production of neuro-toxic APOE fragments. Additionally, P. gingivalis induces neuroinflammation, inflammasome activation, and other immune system multiprotein complexes in the brain that result in neurodegeneration and Aβ plaque formation [27,28]. In animal models, P. gingivalis has been demonstrated to travel to the brain following oral inoculation. Interestingly, in mouse models, Aβ1-42 was found to act as an antimicrobial peptide against P. gingivalis. Aβ1-42 inhibited P. gingivalis by disrupting its cell membrane. Notably, P. gingivalis can be detected in the CSF of AD patients and can thus potentially be used as a biomarker for AD [27]. Other bacteria involved in periodontitis include Aggregatibacter actinomycetemcomitans, Prevotella intermedia, Fusobacterium nucleatum, Tannerella forsythensis, Eikenella corrodens, and Treponema denticola. These various bacteria induce inflammation and thus promote neurodegeneration, though there is some evidence alluding to their presence in the brain [26]. The spirochete Borrelia burgdorferi (B. burgdorferi) is the causative agent of Lyme disease, and has also been linked with AD. B. burgdorferi has been detected in the brains of AD patients and is known for its neurodegenerative effects [29,30]. Additionally, Chlamydia pneumoniae (C. pneumoniae) has been detected in the brains of AD patients and may be another factor driving AD pathology [31]. Infection with Helicobacter pylori (H. pylori) has also been associated with increased risk of AD. Infection with H.pylori has been associated with lower cognitive abilities, as well as increased levels of CSF tau and phosphorylated tau among AD patients [32]. Gut microbiome dysbiosis and altered microbiome composition have been implicated in AD and various other neurodegenerative disorders [33]. The gut microbiome produces lipopolysaccharides, neurotoxins, and microbial amyloid. These bacterial products are involved with amyloid plaque formation, neurofibrillary tangles, and neuroinflammation. The gut microbiome composition is also altered in individuals with AD, with the increased relative abundance of bacteria of the genera Verrucomicrobia and Proteobacteria and decreased abundance of Ruminococcus and Butyricicoccus genera [33]. Interestingly, gut microbiota have also been found in the brains of AD patients [34]. The penetrance of gut bacteria into the brain could represent a potential trigger of amyloidosis, that could occur independently of traditional pathogen infection (Figure 2). This could potentially represent a potential mechanism similar to the association between gingivitis and AD, whereby dysregulation of the microbiome could render the host susceptible to amyloidosis and neurodegeneration.

### 2.3. Other Pathogens and AD

Fungal infection may also play a role in the pathology of AD. Fungal proteins and DNA have been detected in the brains of AD patients [35]. Additional studies have revealed that fungal proteins and DNA can also be detected peripherally and may make suitable biomarkers (see below for further discussion). The protozoan parasite T. gondii, which is thought to infect up to 50% of the world’s population, is known to cause encephalitis and neurological dysfunction. It is thought that T. gondii may be involved with neuroinflammation and olfactory dysfunction in AD pathology [36].

## 3. Pathogen-Based Biomarkers

Based on the above-mentioned studies linking various pathogens with AD and age-related cognitive decline, it may be rational to use biomarkers based on pathogen exposure to assess the risk for developing AD in elderly individuals. In cases of active infection, intervention with antimicrobial treatments may be a suitable method of reducing AD risk, particularly in patients with advanced age. We discuss below a number of biomarkers based on pathogens which have been linked to AD and cognitive decline (Table 1).

### 3.1. Antimicrobial Peptides as Biomarkers in AD

Defensins are a family of disulfide knotted antimicrobial peptides that entrap pathogens as a part of the innate immune system [37]. Moreover, ⍺-Defensins 1 and 2 were shown to be elevated in the blood of AD patients and may make a suitable biomarker for detecting AD status [38]. Another anti-microbial protein that might make a suitable biomarker for AD is lactoferrin. Lactoferrin is an antimicrobial peptide that is present in saliva and correlates with AD status. Saliva samples from amnesiac mild cognitive impairment (aMCI) and AD patients showed decreased levels of lactoferrin when compared with controls, and a significant negative correlation was found between lactoferrin and aMCI and AD patients. These results suggest the potential usage of lactoferrin as a non-invasive salivary biomarker for AD [39]. Tears contain a number of antimicrobial proteins that act as part of the innate immune system. Several proteins present in tears have been shown to be differentially expressed in AD patients and may serve as suitable biomarkers. In particular, changes in the expression of the antimicrobial proteins lipocalin-1, dermcidin, lysozyme-C and lacritin have been reported using tear samples of AD patients [40].

### 3.2. Antibodies as Biomarkers for AD

Antibodies against pathogens associated with AD are readily detectable in blood, and may be a reasonable way of establishing AD risk in the elderly. A number of studies have shown correlations between antibodies against various pathogens and cognitive decline. For example, elevated levels of IgG against Epstein–Barr Virus (EBV) have been shown to correlate with the development of aMCI [41]. The presence of C. pneumoniae in AD patients has been well documented (see discussion above), and IgG and IgA antibodies against C. pneumoniae have been detected in patients with vascular dementia [42]. Additionally, IgG antibodies against HSV-2, CMV, and TOX have been shown to correlate with cognitive decline in individuals over the age of 65 and may also serve as rational biomarkers. While the study by [19] did not find any significant correlation between HSV-1 antibodies and cognitive decline, other groups have reported on the possibility of HSV-1 antibodies as potential biomarkers for AD [43,44]. In a study by Roubaud-Baudron et al., the presence of IgG antibodies against H.Pylori was associated with lower scores on the mini-mental state examination (MMSE) and increased CSF tau levels, among AD patients [32]. AD has also been linked with increased T. gondii IgG antibodies. These antibodies can serve as potential AD biomarkers, given the high prevalence of T. gondii infection globally [36].

### 3.3. Other Potential Biomarkers

Additional pathogens such as fungi may also serve as potential biomarkers in AD. Fungal proteins and DNA have been detected in the CSF for AD patients [45]. Furthermore, fungal polysaccharides, proteins, and DNA have all been detected in blood samples drawn from AD patients [35]. The relationship between the gut microbiome and various neurological diseases has been an area of growing interest in recent years, and may be another option to consider for monitoring the progression of AD. Variation in gut microbiome composition detected from stool samples can also be a preclinical biomarker for AD, given the increased relative abundance of bacteria of the genera Verrucomicrobia and Proteobacteria found in AD. Gut microbiome products such as microbial amyloids and neurotoxin BMAA play a role in neurodegeneration, and can also potentially serve as AD biomarkers [33,46].

## 4. Conclusions

While the presence of pathogens or antibodies against certain pathogens may not directly indicate a positive diagnosis of AD per se, it is important to note that these biomarkers may be appropriate for determining at-risk cohorts of elderly individuals. The potential to identify at risk individuals and administer prophylactic treatments is of great value to the field of AD research. In particular, the administration of antimicrobial treatments (antiviral, antifungal, antibacterial, anti-parasitic) after positive confirmation of an infection carries little risk, and could be neuroprotective. A recent analysis of data from Taiwan’s National Health Insurance Research Database found that treatment with antiherpetic medications was associated with a decreased risk of developing dementia [47]. Furthermore, a clinical trial is currently underway to evaluate the antiviral therapy valacyclovir in the treatment of AD [48].

As it stands, there is currently preclinical evidence to suggest that β-amyloid can directly bind pathogens, and that pathogenic infection can accelerate amyloid pathology in transgenic animal models of AD. Additionally, multiple preclinical studies have demonstrated the neurodegenerative properties of certain pathogens. In terms of clinical data, there are a number of studies providing correlational evidence between the presence of various pathogens and the diagnosis of AD. There is thus a critical missing link between amyloid entrapment of pathogenic microbes, and widespread neurodegeneration. Some insight as to the potential mechanism by which infection may stimulate neurodegeneration can be taken from a recent study examining interferon signaling in response to β-amyloid. Roy and colleagues showed that soluble oligomers interact with nucleic acids (DNA and RNA) or glycosaminoglycans (i.e., heparin), and that these interactions promote the formation of amyloid fibrils. Interestingly, only amyloid fibrils containing nucleic acids promote type 1 interferon response, inflammation, and synaptic loss. Type 1 interferon response was observed across several different transgenic mouse lines, indicating that self-DNA or self-RNA may trigger this response. Additionally, wild type mice that received a hippocampal injection of amyloid fibrils containing RNA showed an inflammatory profile similar to that observed in transgenic models of AD [49]. These findings are very exciting when viewed in the context of other studies, which have shown that amyloid fibrils can form after binding to viral particles such as herpes simplex virus (a double stranded DNA virus). Type 1 interferon response usually occurs as a part of the innate immune response to viral infection; thus, the finding that nucleic acid containing amyloid fibrils stimulates type 1 interferon suggests that Aβ may be an integral component of antiviral defense in the brain. Blocking type 1 interferon response and other immune-related signaling pathways which occur after Aβ entrapment of pathogens may thus be a rational therapeutic strategy in treating AD.

A critical question also becomes whether AD is directly stimulated by one or more pathogens entering the brain, or if AD can occur as a result of dysfunction of Aβ driven anti-viral defenses. Thus, future studies investigating both the function and dysfunction of innate immune responses in the brain will be critical to our understanding and diagnosis of AD. In particular, it is necessary to understand how other factors, such as diet, genetic background, and exposure to environmental toxins may confer susceptibility to neuroinvasion by pathogenic microbes. It is also possible that the reason why so many different pathogens are readily detected in the brains and blood of AD patients is that AD may fundamentally weaken the immune system. Decreased activity or dysfunction of the peripheral immune system may force the innate immune system of the brain to bear a heavy burden when faced with pathogenic infections during AD. Thus, increased reliance on the antimicrobial activity of Aβ during AD may force an already burdened system to a “breaking point”, in which severe neurodeneration is facilitated by excessive amyloidosis, microglial activation, and immune dysfunction in the brain.

There is still a great deal of research that needs to be done to establish a direct causal link between AD and pathogenic infection. In particular, several key areas need to be addressed. One critical consideration is that AD may merely increase susceptibility of infection, thus allowing various pathogens to enter brain and making their detection either a secondary occurrence or an artifact. To address this possibility, it will be critical to examine the brains of patients with familial AD to determine if pathogens can be detected that are not present in age-matched controls. This would help one to better understand if AD fundamentally facilitates pathogenic infections in the brain, which would provide insight as to whether infection is a primary or secondary occurrence in sporadic AD. If no pathogens are present in the brains of patients with familial AD, it might suggest that AD is primarily caused by the brain’s innate immune system behaving in a dysfunctional manner (i.e., amyloid entrapment of host DNA/RNA as opposed to pathogen DNA/RNA). Another critical area which must be addressed is the high detection rate of different pathogens in various cohorts of AD patients. For example, differing studies have found P. gingivalis, LV, or human herpesvirus (as well as other pathogens) in all or nearly all of the brains from AD patients observed in the respective cohorts of each study [17,20,27]. This would imply that, if we were to extrapolate the findings of each individual cohort to the broader AD population, then all AD patients would be expected to present with multiple pathogenic infections simultaneously. Thus, it is crucial to determine if multiple pathogens can indeed be detected within the same brains of AD patients. If multiple pathogens cannot be detected within a single AD brain, it might imply that geographical differences in exposure to various pathogens might cause a specific pathogen to be overrepresented in one particular cohort. This would also suggest the possibility that multiples pathogens might be independently capable of stimulating the same pathogenic processes within AD. While the underlying mechanisms linking specific infections and AD are not explicitly known, the consistent association of various pathogens in AD cannot be ignored. It is thus critical to evaluate the presence of pathogen related biomarkers in elderly individuals, to aid in the construction of an AD risk profile.

## Figures and Tables

**Table 1:** Pathogen-Based Biomarkers in AD. Numerous pathogens have been associated with AD pathogenesis and the onset of cognitive decline. Biomarkers are listed along with their source and relationship to AD.

	Biomarker	Source	Description	Reference
Antimicrobial Peptides				
	⍺-Defensin 1	Blood	Increased in blood of AD patients.	[39]
	⍺-Defensin 2	Blood	Increased in blood of AD patients.	[39]
	Lactoferrin	Saliva	Decreased with AD and aMCI.	[40]
	Lipocalin-1	Tears	Decreased in AD.	[41]
	Dermcidin	Tears	Increased in AD.	[41]
	Lysozyme-C	Tears	Decreased in AD.	[41]
	Lacritin	Tears	Decreased in AD.	[41]
Antibodies				
	IgG against Epstein-Barr Virus	Blood	Correlates with development of aMCI.	[42]
	IgG and IgA against *C. pneumoniae*	Blood	Detectable in patients with vascular dementia.	[43]
	IgG against HSV-2	Blood	Correlates with cognitive decline.	[25]
	IgG against CMV	Blood	Correlates with cognitive decline.	[25]
	IgG against *T. gondii*	Blood	Correlates with cognitive decline.	[25]
	IgM against HSV-1	Blood	Associated with increased risk of AD.	[44,45]
	IgG against *H. Pylori*	Blood	Associated with lower MMSE scores.	[35]
Other				
	Fungal Proteins and DNA	CSF, Blood	Detectable in AD patients.	[36,46]
	Gut Microbiome composition	fecal matter	Correlates to gut dysbiosis and cognitive decline.	[6,47]
	*Porphyromonas gingivalis*	CSF	Identified in 96% of postmortem brain tissue samples of AD patients.	[30]
